# Defining an Abnormal Geriatric Assessment: Which Deficits Matter Most?

**DOI:** 10.3390/cancers15245776

**Published:** 2023-12-09

**Authors:** Anthony Carrozzi, Rana Jin, Susie Monginot, Martine Puts, Shabbir M. H. Alibhai

**Affiliations:** 1 Department of Medicine, Toronto General Hospital, University Health Network, Toronto, ON M5G 2C4, Canada; 2 Temerty Faculty of Medicine, University of Toronto, Toronto, ON M5S 1A8, Canada; 3 Department of Nursing, Princess Margaret Cancer Centre, University Health Network, Toronto, ON M5G 2M9, Canada; 4 Lawrence S. Bloomberg Faculty of Nursing, University of Toronto, Toronto, ON M5T 1P8, Canada

**Keywords:** geriatric assessment, geriatric assessment domains, treatment plan modification, treatment decision making, frailty, older adults, cancer, geriatric oncology, geriatrics, oncology

## Abstract

**Simple Summary:**

The geriatric assessment is used to identify frailty in older cancer patients and guide treatment decision making, but there is no consensus on the definition of what constitutes a globally abnormal assessment. The aim of our study was to propose a definition by determining whether a particular number or constellation of geriatric assessment domains more frequently leads to oncologic treatment plan modification. We found that an abnormal GA may be best defined as one with ≥4/8 abnormalities or abnormalities in the domains of cognition, comorbidities, and falls risk. Our results highlight which domains oncologists should regularly assess in older cancer patients when making treatment decisions. They will also aid cross-study comparisons in geriatric oncology and provide an empirically based frailty definition to be used in study inclusion/exclusion criteria. Finally, our findings will encourage future studies which aim to improve current screening tools for geriatric oncology and/or a simplified version of the geriatric assessment.

**Abstract:**

At present, there is no clear definition of what constitutes an abnormal geriatric assessment (GA) in geriatric oncology. Various threshold numbers of abnormal GA domains are often used, but how well these are associated with treatment plan modification (TPM) and whether specific GA domains are more important in this context remains uncertain. A retrospective review of the geriatric oncology clinic database at Princess Margaret Cancer Centre in Toronto, Canada, including new patients seen for treatment decision making from May 2015 to June 2022, was conducted. Logistic regression modelling was performed to determine the association between various predictor variables (including the GA domains and numerical thresholds) and TPM. The study cohort (n = 736) had a mean age of 80.7 years, 46.1% was female, and 78.3% had a VES-13 score indicating vulnerability (≥3). In the univariable analysis, the best-performing threshold number of abnormal domains based on area under the curve (AUC) was 4 (AUC 0.628). The best-performing multivariable model (AUC 0.704) included cognition, comorbidities, and falls risk. In comparison, the multivariable model with the sole addition of the threshold of 4 had an AUC of 0.689. Overall, an abnormal GA may be best defined as one with abnormalities in the domains of cognition, comorbidities, and falls risk. The optimal numerical threshold to predict TPM is 4.

## 1. Introduction

The population of older adults worldwide is growing rapidly. By 2050, it is expected that the number of people aged 65 years and older will nearly triple in size in comparison to 2010, increasing from an estimated 524 million to almost 1.5 billion [1]. In general, older patients are more complex than their younger counterparts due to the presence of a higher number of comorbidities, polypharmacy, and functional impairments, among many other factors [2]. The heterogeneity of aging further complicates this picture as clinicians are not able to take a standardized approach driven by chronological age. This complexity and heterogeneity is even more pronounced in older adults with cancer, given the diversity of sites, subtypes, and available treatments.

For these reasons, there exists a growing need for cancer care tailored to older adults. Numerous centers worldwide have opened geriatric oncology clinics or comprehensive care programs to address this [1]. Geriatric oncology practice is rooted in the geriatric assessment (GA), which seeks to achieve two main goals: (1) to tailor oncologic treatment decisions to the patient’s ability to tolerate treatment, and (2) to implement interventions aimed at optimizing the patient’s health status, improving their ability to tolerate treatment [3]. Numerous randomized controlled trials have demonstrated that GA and intervention are able to achieve these goals, reducing falls, polypharmacy, and treatment toxicity, as well as improving quality of life and healthcare delivery [4,5,6].

Although the utility and importance of GA in geriatric oncology has been demonstrated, one of its major limitations is that it is often too time consuming and resource-intensive to be carried out by the average clinician. Some have proposed using a screening tool for frailty (which may predict outcomes of the GA) as a more feasible practice to implement in oncology at the present time. A systematic review by Hamaker et al. (2012) aimed to assess the sensitivity and specificity of seven available frailty screening tools in older adults with cancer and found that, even for the tool with the highest sensitivity, the negative predictive value was only around 60% [7]. Thus, they concluded that GA remains the ideal method of capturing frailty in geriatric oncology as the available screening tools have insufficient discriminative power.

Though GA is the present gold standard, there is no universal, objective, and evidence-based definition of what constitutes an abnormal GA in geriatric oncology. In the literature, a threshold number of abnormal GA domains (ranging from 1–4) is often used to define an abnormal GA (i.e., one that indicates frailty, which is linked to a greater risk of adverse outcomes and the need to modify cancer treatment) [8]. In 2015, Handforth et al. published a review which aimed to establish the prevalence and outcomes of frailty in older cancer patients [9]. Sixteen of the 20 studies they examined used GA for diagnosis of frailty, with the median number of domains assessed being 7 (range: 3–9). Eight studies dichotomized patients as either frail or fit, with four using a threshold of 2 abnormal GA domains, two using a threshold of 3, one using a threshold of 2 or cognitive impairment only, and one using no threshold number but requiring two independent physicians to diagnose frailty. The other eight studies categorized patients as fit, pre-frail, or frail and had great variation in the thresholds used to differentiate each category as well, ranging from 1 to 3 abnormal domains for pre-frail and 1–4 for frail [9]. This lack of consensus in defining frailty based on GA inevitably makes comparisons across studies in geriatric oncology very challenging.

Recognizing the need outlined above, Bréchemier et al. (2019) set out to establish a consensual and operational definition of frailty for geriatric oncology by comparing GA data with Fried’s phenotype of frailty, a common measurement of frailty in geriatrics [8]. They found that Fried’s phenotype of frailty is associated with more impaired geriatric domains and a threshold of 4 abnormal domains could be used to detect frailty in a GA. However, they acknowledged that a limitation of their study was that they did not evaluate the impact of each GA domain on frailty risk (a previous study showed different weightings for each domain based on mortality risk [10]).

Thus far, we have cited a review which demonstrated the use of arbitrary GA-domain thresholds to define frailty and a study which correlated GA domains with another frailty framework. Despite this, and perhaps more fundamentally important, it is not well established whether having a specific number of abnormal domains more frequently leads to treatment plan modification (TPM), one of the main goals of the GA stated above, or if particular domains have a greater impact on TPM than others. We identified 10 studies that examined the relationship between GA domains and TPM to some extent. In most, the primary focus was not on examining this relationship and none aimed to use this relationship to define an abnormal GA [11,12,13,14,15,16,17,18,19,20]. Functional status, nutrition, cognition, comorbidities, and falls risk were the only GA domains found to be statistically significantly associated with TPM in multivariable analysis in at least one of these studies (Supplementary Table S1). Other studies have demonstrated that impairments in many of these domains are predictive of important clinical outcomes which would be expected to modify the oncologic treatment plan (e.g., mortality, postoperative complications, and systemic treatment toxicity) [21]. As such, there remains a need to directly explore the relationship between abnormal GA domains and TPM and incorporate the results into the definition of an abnormal GA.

Considering the gaps identified above, the primary objectives of this study were: (1) to determine how well the current definitions of an abnormal GA are able to predict TPM following GA, and (2) to identify particular GA domains associated with TPM.

## 2. Materials and Methods

We conducted a retrospective review of the Older Adults with Cancer Clinic (OACC) database at the Princess Margaret Cancer Centre in Toronto, Canada. At the OACC, a GA is performed by a geriatric oncology nurse and physician, as described previously [22]. In accordance with the American Society for Clinical Oncology’s (ASCO) geriatric oncology guidelines (2018), the domains of cognition, comorbidities, falls risk, functional status, medication optimization, mood, nutrition, and social supports are assessed (Supplementary Table S2) [23]. All new patients seen from 22 May 2015 to 10 June 2022 for reasons related to treatment decision making who had a proposed oncologic treatment plan and received a complete GA were included. Demographic variables (age; sex; Vulnerable Elders Survey-13 (VES-13); and Cancer and Aging Research Group (CARG) chemotoxicity risk), oncologic variables (disease site and stage; treatment stage and intent; and current, proposed, and final treatment), GA domains (listed above), and treatment impact were extracted from the clinic database. Treatment impact is determined on an ongoing basis by an expert, non-blinded geriatric oncologist (S.M.H.A.). Following a patient’s visit to the OACC, the results of the GA and the OACC’s recommendations are sent to the referring physician. The referring physician then devises a final treatment plan for the patient. S.M.H.A. reviews the actual treatment received by the patient and classifies it as “increased in intensity”, “unchanged”, “decreased in intensity”, “changed to supportive care only”, “deferred”, or “other”. The outcome of TPM was dichotomized as modification (consisting of final treatment plans designated as “increased in intensity”, “decreased in intensity”, “changed to supportive care only”, or “deferred”) versus no modification (consisting of final treatment plans designated as “unchanged”). Note that patients with final treatment plans designated as “other” were excluded from our cohort. Descriptions of each type of treatment plan impact are included in Supplementary Table S3.

### 2.1. Data Checking

To optimize the quality of the extracted data, a check for missingness in the entire dataset and a check for the accuracy of a 10% subsample (74 patients) were completed. All missingness that was not identified in previous data audits (prior to this study) was reconciled from the patients’ charts and the percent error after reviewing a subsample of the patients’ charts was acceptable at <0.5%.

### 2.2. Statistical Analyses

To determine each predictor variable’s association with TPM, univariable and multivariable analyses (via binary logistic regression modelling) were conducted using the Statistical Package for the Social Sciences (SPSS) version 28.0.1.1. Age (continuous), sex, frailty (VES-13) score, and treatment intent were included in all multivariable models based on clinical relevance (except in cases of collinearity; see Supplementary Methods), regardless of their statistical significance in univariable analysis. Area under the curve (AUC) was calculated for all models and an odds ratio (OR) with a 95% confidence interval (CI) was calculated for all variables. The Hosmer–Lemeshow statistic was also calculated for all multivariable models. The *p*-value for predictor candidacy in multivariable analysis was set at 0.10 and the *p*-value for the significance was set at 0.05. In multivariable analysis, 14 different models were built: one that included all candidate GA domains from the univariable analysis (n = 1), one that included the threshold number of 4 abnormal domains (threshold selected based on the univariable threshold model with the highest AUC) (n = 1), one for each candidate GA domain (n = 6), and one for each candidate GA domain plus the best-performing threshold number of the abnormal domains from univariable analysis (n = 6). No adjustments were made for multiple significance testing [24].

We took three main steps to address possible collinearity amongst the variables in our models. First, we built multiple multivariable models with fewer variables (e.g., single GA domain alone, single GA domain and threshold number of abnormal domains, all GA domains together, etc.). Second, we looked at collinearity by Spearman correlation (correlation coefficient ≥0.5 being concerning for collinearity) and by observing changes in the beta coefficient of suspected collinear pairs when consecutively added into multivariable models together (≥10% change being concerning for collinearity). Third, we calculated the variance inflation factor (VIF) for all variables in all multivariable models (VIF ≥5 being concerning for collinearity). Additional details are provided in Supplementary Methods.

Finally, given that the VES-13 is not commonly used in the oncology setting globally at present, we decided to perform sensitivity analyses excluding VES-13 from each of our models (note that, due to the collinearity checks described above, VES-13 was already excluded from certain models prior to these sensitivity analyses being performed). Our goal was to identify models whose AUC increased by 0.02 units or more and variables that became significantly associated with TPM (as well as associations which previously were significant and became insignificant) following the removal of VES-13. Those models for which the former occurred would replace their counterpart in the reporting of our main multivariable analysis results.

## 3. Results

### 3.1. Characteristics of Study Cohort

**Figure 1:**
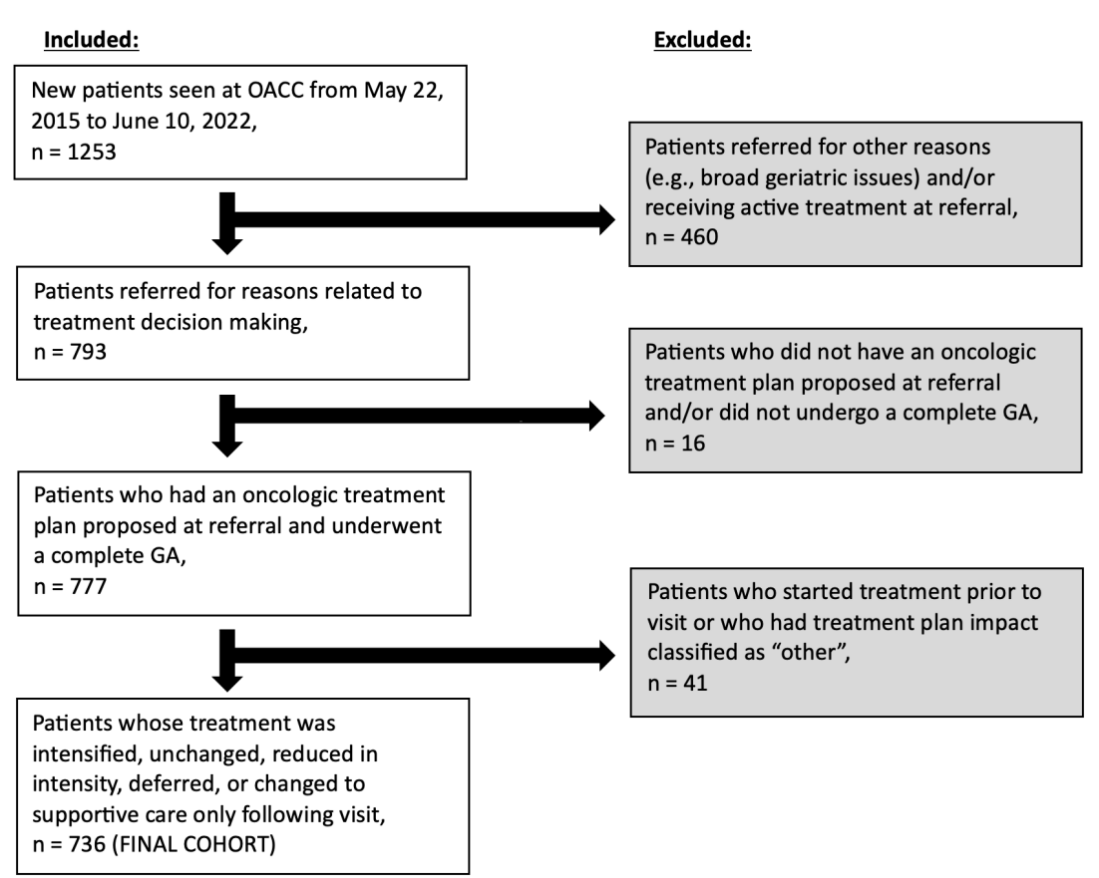
Flow chart illustrating patient cohort selection. Note: “OACC” = Older Adults with Cancer Clinic” and “GA” = geriatric assessment.

Figure 1 outlines the process for patient selection in our study. A total of 1253 new patients were seen for reasons related to treatment decision making at the OACC from 22 May 2015 to 10 June 2022 and our final cohort included 736 of these patients.

Table 1 summarizes the baseline demographic and oncologic characteristics of the study cohort. Overall, the cohort included 736 patients with a mean age of 80.7 years (ranging from 61 to 100 years). Just under half (46.1%) were female and 78.3% had a VES-13 score indicating vulnerability (≥3). Of the patients who were assigned a CARG toxicity risk score (n = 412), 49.3% scored moderate- or high-risk for chemotoxicity. The most frequent disease sites were gastrointestinal (GI) at 27.4%, head and neck (19.8%), genitourinary (GU) (17.7%), lymphoma (10.2%), and gynecologic (9.9%). Sixty-two percent of the sample had a curative or neo/adjuvant treatment intent.

**Figure 2:**
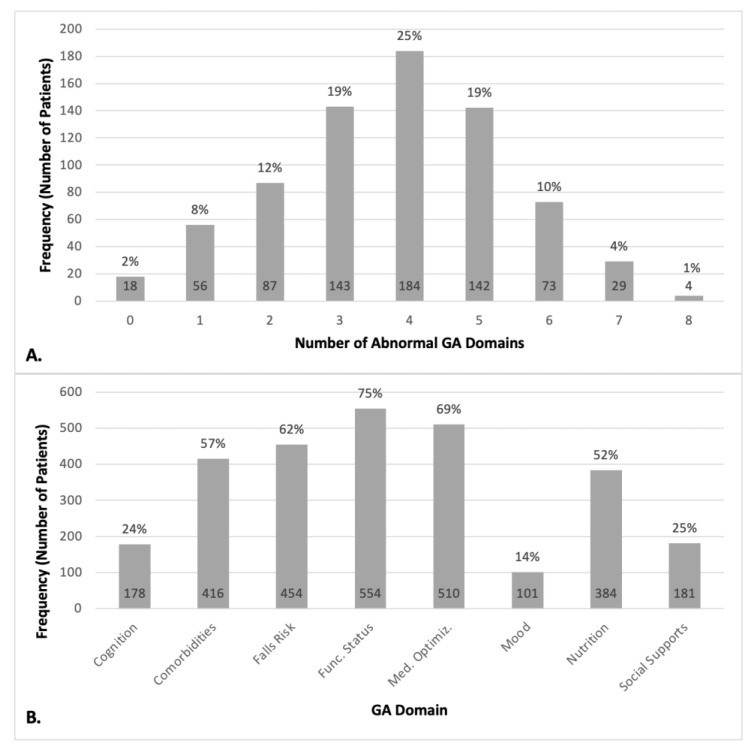
Frequency of abnormalities in the geriatric assessment (GA) domains in the study cohort, by number (panel (**A**)) and type (panel (**B**)). Note: “Func. Status” = functional status and “Med. Optimiz.” = medication optimization.

Panel A of Figure 2 shows that the most frequent number of abnormal domains in our cohort was four at 25%, followed by three (19.4%), five (19.3%), two, (11.8%), six (9.9%), one (7.6%), seven (3.9%), zero (2.4%), and eight (0.5%). Panel B of Figure 2 shows the distribution of these deficits across the GA domains; functional status was the most common at 75.3%, followed by medication optimization (69.3%), falls risk (61.7%), comorbidities (56.5%), nutrition (52.2%), social supports (24.6%), cognition (24.2%), and mood (13.7%).

**Figure 3:**
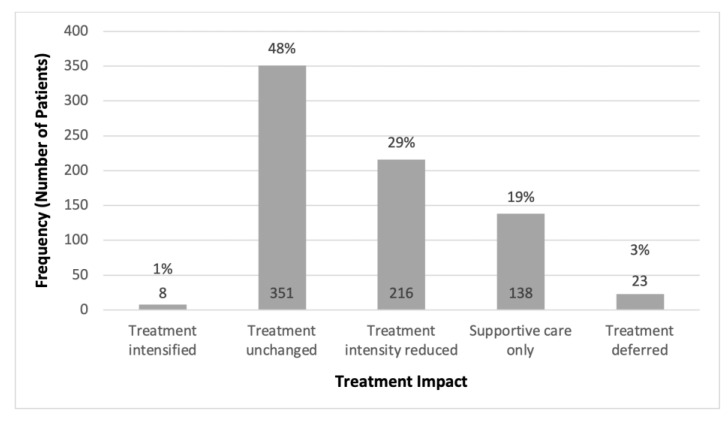
Frequency of different impacts of the geriatric assessment on the oncologic treatment plan.

Figure 3 describes the impact that GA had on the treatment plan after patients were seen at the OACC. The most common was no change in treatment for 47.7% of patients, followed by a reduction in treatment intensity (29.3%) and change to supportive care only (18.8%). Treatment deferral occurred in 3.1% of the patients and treatment intensification in only 1.1%.

### 3.2. Univariable Analysis

Table 2 displays a selection of key results from our univariable analysis, which examines the relationship between each predictor variable individually and TPM. The complete results for all variables can be found in Supplementary Table S4.

Age, VES-13 score, and CARG toxicity risk were the demographic variables significantly associated with TPM. In terms of oncologic variables, treatment stage (overall), disease stage (overall and each stage), and treatment intent (overall and curative or neo/adjuvant) were significantly associated with TPM.

All of the GA domains, except medication optimization and social supports, were significantly associated with TPM in univariable analysis (i.e., cognition, comorbidities, falls risk, functional status, mood, and nutrition). The total number of abnormal domains was also significantly associated with TPM (overall, five, six, and seven).

Finally, the best performing threshold number of abnormal domains based on the AUC was 4 (AUC 0.628) (note that, for this reason, the threshold of 4 was selected as the threshold to be used in the multivariable analysis and so any references to “the threshold” without a numerical specification from this point forward refer to the threshold of 4). All threshold numbers, with the exception of 1, 7, and 8, were significantly associated with TPM.

### 3.3. Multivariable Analysis

As described above, 14 different multivariable models were constructed (key results in Table 3, white cells; complete results in Supplementary Table S5). All models contained age, sex, VES-13 score, and treatment intent as predictor variables, except in cases of collinearity (Supplementary Methods). They all had good calibration, with Hosmer–Lemeshow *p*-values > 0.10 (Supplementary Table S5), and no additional collinearity was detected (all VIFs were <2).

Model 1 additionally contained five candidate GA domains from univariable analysis (functional status was a candidate based on *p*-value but was excluded due to collinearity with falls risk). The AUC of this model was the highest of all the models in our study at 0.704. The predictor variables independently associated with TPM were age (OR 1.04 per year, 95% CI 1.02–1.07), treatment intent (curative or neo/adjuvant; OR 0.58, 95% CI 0.41–0.82), cognition (OR 1.67, 95% CI 1.13–2.47), comorbidities (OR 1.90, 95% CI 1.36–2.65), and falls risk (OR 1.96, 95% CI 1.40–2.76).

In Model 2, the threshold of 4 abnormal domains was included. This model had an AUC of 0.689. Age (OR 1.03 per year, 95% CI 1.00–1.05), VES-13 score (OR 2.01, 95% CI 1.31–3.09), treatment intent (curative or neo/adjuvant; OR 0.56, 95% CI 0.40–0.77), and the threshold of 4 (OR 2.29, 95% CI 1.64–3.20) were independently associated with TPM.

Models 3–8 each contained a single GA domain. The AUCs of these models ranged from 0.650 (Model 7 (mood)) to 0.679 (Models 3 (cognition) and 4 (comorbidities)). In all models, except Models 7 (mood) and 8 (nutrition), the predictor GA domain was independently associated with TPM.

Models 9–14 each contained the threshold of 4 and a single GA domain. The AUCs of these models ranged from 0.685 (Model 13 (threshold + mood)) to 0.700 (Model 9 (threshold + cognition)). The AUC of Model 10 (threshold + comorbidities) was very close to Model 9 at 0.699. In all models, the threshold of 4 abnormal domains was independently associated with TPM; however, Models 9 and 10 were the only ones in which the included GA domain also demonstrated this association.

In terms of the trends observed across all models in the magnitude of effect of particular variables, the largest positive effects were seen with VES-13 score and the threshold. VES-13 score and the threshold of 4 had statistically significant ORs ≥ 1.90 in all models in which they were included, indicating that individuals with an abnormal VES-13 score (≥3) and/or at least 4 abnormal GA domains have approximately twice the chance of receiving a TPM compared to those with a normal score (<3) and/or fewer than 4 abnormal domains, respectively. On the other hand, the variable with the largest negative effect on TPM was the treatment intent (curative or neo/adjuvant). It had statistically significant ORs < 0.60 in all models, indicating that individuals who are designated as curative or neo/adjuvant have nearly half the chance of receiving a TPM compared to those who are designated as palliative.

As for the magnitude of effect of single variables (excluding the standard predictors) in particular models, the largest observed were ORs in the 1.90–2.60 range for the following variables (descending order by OR): functional status in Model 6, the threshold in all models where it was present, falls risk in Models 5 and 1, cognition in Model 3, and comorbidities in Model 1.

### 3.4. Sensitivity Analysis

In sensitivity analysis, all models had lower AUCs (ranging from 0.005 units lower for Model 11 to 0.031 units lower for Model 7) when excluding VES-13 (key results in Table 3, grey cells; complete results in Supplementary Table S6). This suggests that the VES-13 adds some predictive value to this definition which is not captured by age, sex, treatment intent, the GA domains, and the threshold. As for the variables that became significantly associated with TPM, there were relatively few of interest: age in Models 3–5, 7, and 8; mood in Model 7; and falls risk in Model 11. No variables that previously had a significant association with TPM had an insignificant association after removing VES-13. For these reasons, no models from our sensitivity analysis replaced their counterparts in the reporting of our main multivariable analysis results.

## 4. Discussion

We attempted to identify features of an abnormal GA that were associated with TPM, including both numerical thresholds (numbers of abnormal domains) and specific GA domains. Our results suggest that an abnormal GA may be best defined as one with abnormalities in the domains of cognition, comorbidities, and falls risk. In terms of a strictly numerical threshold, a GA may be best defined as abnormal if at least 4 of 8 domains are abnormal. When at least 4 GA domains are abnormal, abnormalities in cognition and comorbidities appear to improve our ability to predict TPM. Overall, models using specific GA-domain deficits performed similarly to threshold-based models for the outcome of TPM. To our knowledge, this is the first study which aims to define an abnormal GA based on GA-domain deficits associated with TPM.

In terms of how our results compare to the existing body of related literature, there is both agreement and disagreement. Functional status, nutrition, cognition, comorbidities, and falls risk were the GA domains found to be associated most frequently with TPM in the multivariable analysis in previous studies (Supplementary Table S1) [11,12,13,14,15,16,17,18,19,20]. We saw this association for all of these variables except functional status and nutrition. This discrepancy may have occurred for a variety of reasons. First of all, these studies were largely conducted in European centers [11,12,13,14,15,17,18,19] and had smaller sample sizes (the largest study [12] with n = 571). Second, there were differences in the processes used to deliver the GA and the way each GA domain was assessed between these studies and with our own. For example, the results by Girre et al. (2008) are based on a 10-min GA screening questionnaire as opposed to the comprehensive GA used in our study [11]. In the same study, Eastern Cooperative Oncology Group Performance Status (ECOG-PS) was used in the functional status assessment and the four-item Geriatric Depression Scale (mini-GDS) was used in the mood assessment [11]. Third, we considered that rarity of abnormality in the domain may contribute to the effects of these variables being obscured, but functional status was the most frequent abnormal domain (75%) and nutrition was abnormal in over half of our cohort (52%; higher than the percentage of patients with abnormal cognition, 24%, and only slightly lower than those with abnormalities in comorbidities, 57%, and falls risk, 62%). Fourth, we looked at functional status and nutrition in isolation. We wondered if, despite our numerous checks, collinearity with falls risk may be preventing an independent association between functional status and TPM from being observed in multivariable analysis. Thus, we compared the raw numbers of cases where there was discordance between an abnormality/normality in functional status and falls risk (with the thought that low numbers of discordant cases could indicate collinearity). This was not observed as a similar number of patients with an abnormal functional status had a normal falls risk as did those with a normal functional status. As for nutrition, we postulate that different disease sites may have differing effects on nutrition (abnormalities potentially being more common and impactful in sites like head and neck and gastrointestinal) [25,26]. Thus, the relative proportion of these disease sites represented in the related literature could impact the association established with TPM.

In terms of how our results compare to previously studied numerical threshold definitions of an abnormal GA, the one study which examined the association of Fried’s phenotype of frailty with the number of impaired GA domains found 4 to be the ideal threshold [8]. This similarity lends support to our results.

We anticipate that the results in the present study may have both clinical and research implications. On the clinical side, this definition emphasizes that oncologists should regularly assess older patients in the GA domains of cognition, comorbidities, and falls risk. Doing so may enable them to more accurately and confidently identify patients who would benefit from a GA. In many clinical settings, this may be more feasible than assessing all eight major GA domains. Similarly, it will increase oncologists’ confidence in independently modifying treatment (i.e., if GA is not possible) as it provides a precedent for TPM when a specific number and/or constellation of domains is/are impaired. On the research end, it will contribute to addressing the need for a universal, objective, and evidence-based definition of an abnormal GA, improving cross-study comparisons in geriatric oncology. This definition may change the way we develop future screening tools for geriatric oncology as we are defining abnormality based on an outcome (TPM) rather than an arbitrary number. For the same reason, this definition may be used as an inclusion/exclusion criterion for potential studies which seek to characterize older cancer patients’ frailty in a manner that can be empirically defended. At present, common geriatric screening tools, like the Geriatric-8 (G-8) and VES-13, do not focus on multiple domains nor do they assess all three domains that we have highlighted. Including these three domains may improve screening tool performance. Finally, this definition may encourage future studies which attempt to simplify the GA into a more rapid assessment (potentially a form that could be conducted by clinicians who do not have specialized training in geriatrics).

In addition to the future research directions noted above, we hope that our findings will encourage others who have access to large geriatric oncology datasets to replicate our study. Multicenter studies, studies in other jurisdictions and healthcare systems, studies which examine single disease sites, and studies which explore outcomes other than TPM would add great value to this definition of an abnormal GA and help to establish new definitions in more specific contexts. Though most existing toxicity prediction tools have been developed in heterogenous populations (including multiple disease sites, stages, and treatment regimens), there are cases where tools developed for a specific disease site perform better than their generalized counterpart. For example, there was a version of the CARG developed in 2021 for early-stage breast cancer (BC) patients called the CARG-BC [27]. It demonstrated a superior ability to predict grade 3–5 chemotoxicity in patients with early-stage breast cancer compared to the generalized CARG tool (AUC = 0.69 vs. AUC = 0.56). The authors found that cancer stage, regimen, planned treatment duration, and liver function were important predictors for the CARG-BC model but not for the generalized model. Thus, they emphasize the importance of future studies that focus on specific cancer sites. Additionally, with the emergence of geriatric subspecialities outside of oncology, the GA will be applied to measure frailty in different clinical contexts. For example, in geriatric cardiology, frailty has become a measure used to quantify risk among patients with aortic stenosis who are being considered for interventions such as transcatheter aortic valve replacement (TAVR) [28,29]. Understanding which and/or how many GA domains are more likely to predict a successful TAVR in an older patient would enable a more systematic application of the GA in this setting. Similarly, in geriatric nephrology, using the GA to measure frailty may enable clinicians to better predict outcomes for potential hemodialysis patients [30,31].

Our study possessed many strengths, as outlined above, including a large dataset composed of a heterogeneous population of patients and the fact that it links the concept of an abnormal GA with a clinically relevant outcome (TPM). However, certain limitations exist which one should be aware of. Even though we had a relatively large sample size (n = 736), it was not possible to stratify our analyses by disease site due to the large number of sites represented in our cohort. The study’s retrospective design did not permit us to rectify this problem by increasing the sample size to a level that would enable such analyses. Additionally, since our sample was derived from a single academic geriatric oncology clinic in a universal healthcare system, our results may be less relevant in community settings and/or other jurisdictions. A related limitation is that, since our study aims to define abnormal rather than normal, it assumes unidirectional TPM (i.e., de-intensification). This is reasonable in our context, as only eight patients in our cohort received treatment intensification following GA; however, if there is an attempt to replicate this study in other populations, it may be important to stratify TPM by the direction of the change. Moreover, to limit potential confounding, we systematically examined all available studies looking at similar relationships with TPM and included all relevant predictor variables in our analyses. However, there always exists the possibility of unmeasurable confounders which could influence the results of such retrospective observational studies. Finally, we did not adjust for multiple significance testing, which may increase the risk of false positives. While adjustment is widely accepted for practice-changing clinical trials, it is more controversial for observational studies [24]. Instead, we were transparent about our modelling, reported confidence intervals for all estimates, attempted model simplification where possible, and performed sensitivity analyses to explore the robustness of our findings.

## 5. Conclusions

In summary, our study addresses the question of which of the current arbitrary thresholds used to define an abnormal GA actually impacts the oncologic treatment plan (≥4 abnormal domains) and identifies particular GA-domain deficits associated with TPM (cognition, comorbidities, and falls risk). Both approaches are similarly predictive of TPM. The results may influence both clinical practice and future research work. It is our desire that they will further promote the use and importance of the GA, as well as emphasize the need and potential feasibility of a screening tool designed specifically for geriatric oncology.

## Figures and Tables

**Table 1:** Baseline demographic and oncologic characteristics of the study cohort (n = 736).

Demographic Characteristics		Mean (Range) or N (%)
Age, y		80.7 (61–100)
Sex	Female	339 (46.1%)
VES-13 Score	≥3 (Vulnerable)	576 (78.3%)
CARG Toxicity Risk	Low	49 (6.7%)
	Moderate	182 (24.7%)
	High	181 (24.6%)
	N/A	324 (44.0%)
Oncologic Characteristics		N (%)
Disease Site	GI	202 (27.4%)
	Head and Neck	146 (19.8%)
	GU	130 (17.7%)
	Lymphoma	75 (10.2%)
	Gynecologic	73 (9.9%)
	Other	110 (14.9%)
Disease Stage	Locally advanced	245 (33.3%)
	Localized	227 (30.8%)
	Metastatic	170 (23.1%)
	Hematologic	85 (11.5%)
	Unknown	9 (1.2%)
Treatment Intent	Curative and Neo/adjuvant	452 (61.4%)
	Palliative	263 (35.7%)
	Unknown	21 (2.9%)

Note: “VES-13” = Vulnerable Elders Survey-13, “CARG” = Cancer and Aging Research Group, “GI” = gastrointestinal, and “GU” = genitourinary.

**Table 2:** Results of the univariable analysis examining the relationship between select predictor variables and treatment plan modification.

Predictor Variable		OR (95% CI)	AUC
Age (Continuous)		1.03 (1.01–1.06)	0.563
Sex (Male)		0.90 (0.68–1.21)	0.513
VES-13 Score (Abnormal, >3)		3.35 (2.29–4.91)	0.597
CARG Toxicity Risk	Low (reference)	N/A	0.649
	Moderate	2.10 (1.09–4.05)	
	High	5.86 (2.97–11.56)	
	N/A	1.35 (0.72–2.52)	
Treatment Intent	Palliative (reference)	N/A	0.590
	Curative or Neo/Adjuvant	0.48 (0.35–0.66)	
	Unknown	1.46 (0.55–3.89)	
Cognition (Abnormal)		2.24 (1.57–3.19)	0.572
Comorbidities (Moderate/High)		2.16 (1.61–2.91)	0.594
Falls Risk (Increased)		2.49 (1.83–3.37)	0.606
Functional Status (Impaired Physical Performance and/or IADLs)		2.69 (1.89–3.83)	0.588
Medication Optimization (Potential for Optimization)		1.10 (0.81–1.51)	0.511
Mood (Depressed)		1.62 (1.05–2.50)	0.529
Nutrition (At-Risk/Malnourished)		1.39 (1.04–1.85)	0.541
Social Supports (Vulnerable/None)		1.10 (0.79–1.54)	0.509
Thresholds (binary; max. 8 domains)	≥1 Abnormal Domains	2.24 (0.83–6.02)	0.509
	≥2 Abnormal Domains	3.31 (1.82–5.23)	0.548
	≥3 Abnormal Domains	2.88 (1.99–4.17)	0.588
	≥4 Abnormal Domains	2.94 (2.17–3.99)	0.628
	≥5 Abnormal Domains	2.62 (1.90–3.61)	0.604
	≥6 Abnormal Domains	2.37 (1.52–3.69)	0.551
	≥7 Abnormal Domains	1.87 (0.90–3.92)	0.513
	8 Abnormal Domains	0.91 (0.13–6.50)	0.500

Note: “VES-13” = Vulnerable Elders Survey-13, “CARG” = Cancer and Aging Research Group, “IADLs” = instrumental activities of daily living, “OR” = odds ratio, “CI” = confidence interval, and “AUC” = area under the curve. The “N/A” category is for patients who were not assigned a CARG risk score as chemotherapy was not planned.

**Table 3:** Results of the multivariable analysis examining the relationship between select predictor variables and treatment plan modification in the indicated models.

No.	AUC	Cognition (Abnormal)	Comorbidities (Moderate/High)	Falls Risk (Increased)	Functional Status (Impaired Physical Performance and/or IADLs)	Mood (Depressed)	Nutrition (At-Risk/Malnourished)	Threshold (≥4 Abnormal Domains)
1	0.704	1.67 (1.13–2.47)	1.90 (1.36–2.65)	1.96 (1.40–2.76)	*	1.36 (0.84–2.19)	1.31 (0.94–1.82)	-
2	0.689	-	-	-	-	-	-	2.29 (1.64–3.20)
	0.682	-	-	-	-	-	-	2.81 (2.06–3.84)
3	0.679	1.96 (1.35–2.83)	-	-	-	-	-	-
	0.657	2.16 (1.50–3.11)	-	-	-	-	-	-
4	0.679	-	1.88 (1.38–2.58)	-	-	-	-	-
	0.659	-	2.12 (1.56–2.87)	-	-	-	-	-
5	0.678	-	-	1.97 (1.42–2.74)	-	-	-	-
	0.669	-	-	2.42 (1.77–3.31)	-	-	-	-
6	0.662	-	-	-	2.57 (1.79–3.70)	-	-	-
7	0.650	-	-	-	-	1.45 (0.91–2.23)	-	-
	0.619	-	-	-	-	1.58 (1.01–2.48)	-	-
8	0.660	-	-	-	-	-	1.24 (0.91–1.68)	-
	0.635	-	-	-	-	-	1.33 (0.99–1.80)	-
9	0.700	1.51 (1.02–2.23)	-	-	-	-	-	2.10 (1.47–3.00)
	0.693	1.50 (1.01–2.20)	-	-	-	-	-	2.56 (1.83–3.59)
10	0.699	-	1.54 (1.11–2.15)	-	-	-	-	1.99 (1.40–2.83)
	0.692	-	1.58 (1.14–2.20)	-	-	-	-	2.41 (1.73–3.36)
11	0.691	-	-	1.40 (0.94–2.06)	-	-	-	1.91 (1.28–2.84)
	0.686	-	-	1.51 (1.02–2.24)	-	-	-	2.20 (1.49–3.23)
12	0.689	-	-	-	1.51 (0.98–2.33)	-	-	2.35 (1.62–3.40)
13	0.685	-	-	-	-	1.16 (0.72–1.86)	-	2.20 (1.56–3.11)
	0.677	-	-	-	-	1.17 (0.73–1.87)	-	2.66 (1.92–3.69)
14	0.689	-	-	-	-	-	0.97 (0.69–1.34)	2.31 (1.63–3.29)
	0.683	-	-	-	-	-	0.95 (0.69–1.32)	2.84 (2.04–3.96)

Note: “AUC” = area under the curve, “*” = variable excluded from model due to collinearity concerns, and “-” = variable not present in model. The values present in each variable column are odds ratios, along with their 95% confidence intervals in parentheses. All models were adjusted for age, sex, Vulnerable Elders Survey-13 (VES-13) score, and treatment intent. Results of the sensitivity analysis (excluding VES-13 from each model) are present in the grey rows. Complete results from the multivariable analysis can be found in Supplementary Tables S5 and S6.

## Data Availability

Restrictions apply to the availability of these data. Data were obtained from the University Health Network and are available from the authors with permission of the Research Ethics Board of the University Health Network.

## Supplementary Materials

The following supporting information can be downloaded at: https://www.mdpi.com/article/10.3390/cancers15245776/s1, Supplementary Methods; Supplementary Table S1: Frequency of reports on statistically significant associations between relevant predictor variables and treatment plan modification in univariable and multivariable analyses in similar studies; Supplementary Table S2: Descriptions of the evaluation methods for each GA domain and what constitutes an abnormal designation in our study; Supplementary Table S3: Descriptions of the types of treatment plan impact; Supplementary Table S4: Complete results of univariable analysis examining the relationship between various predictor variables and treatment plan modification; Supplementary Table S5: Complete results of multivariable analysis examining the relationship between various predictor variables and treatment plan modification; Supplementary Table S6: Complete results of multivariable analysis examining the relationship between various predictor variables and treatment plan modification (sensitivity analysis, excluding VES-13 score).
