# Supplementary material for: Defining an Abnormal Geriatric Assessment: Which Deficits Matter Most?
Source: Cancers (Basel). 2023 Dec 9;15(24):5776. doi: 10.3390/cancers15245776 (PMC10742229; doi:10.3390/cancers15245776)
Supplement: Supplementary file 1 [file cancers-15-05776-s001.zip › cancers-2708279-supplementary.pdf]

Supplementary Methods

When commencing data analysis, we suspected disease stage and treatment intent would be collinear as they are clinically similar parameters. We confirmed collinearity and decided to only include treatment intent in multivariable analysis as it had a better univariable AUC and it was more relevant clinically (as, unlike disease stage, it does not exclude patients with hematologic malignancies).

Next, we performed a Spearman correlation including all candidate (p-value <0.10) variables from univariable analysis. We found collinearity between VES-13 Score and functional status, functional status and falls risk, functional status and the threshold, and falls risk and the threshold. All of these collinear relationships were confirmed by observing changes in the beta coefficient when trialling different combinations. The models affected by these collinear relationships included Models 1, 6, 11, and 12. In Model 1, we decided to exclude VES-13 Score (rather than functional status) as it is not commonly used in the oncology setting globally. We then excluded functional status (rather than falls risk) from this model as it had a lower odds ratio. In Model 6, we decided to exclude VES-13 Score (rather than functional status) as the main goal of this model was to examine the association of functional status with treatment plan modification (TPM) on its own (while controlling for the standard variables included in every multivariable model). In Model 11, we did not make adjustments for collinearity as removing falls risk or the threshold would make this model identical to Models 2 or 5, respectively. As such, we decided to report this model in our paper for the sake of completeness (i.e., including all models with a single GA domain and the threshold), but have flagged it for collinearity. In Model 12, we decided to exclude VES-13 Score (rather than functional status) for the same reasons identified above, but we did not remove the threshold for the sake of completeness (as in Model 11).

Supplementary Table S1. Frequency of reports of statistically significant associations between relevant predictor variables and treatment plan modification in univariable and multivariable analyses in similar studies.

Variable	Frequency	
	Univariable	Multivariable
Functional Status	9 (12,13, 14, 15, 16, 17, 18, 19, 20)	7 (12, 14, 15, 17, 18, 19, 20)
Nutrition	6 (11, 12, 14, 15, 18, 19)	3 (12, 14, 18)
Cognition	4 (12, 14, 18, 19)	2 (12, 18)
Age	3 (12, 18, 20)	2 (12, 20)
Falls Risk	4 (14, 15, 16, 19)	1 (19)
Comorbidity	3 (13, 14, 19)	1 (19)
Depression	3 (11, 14, 15)	0
Medication Optimization	2 (14, 19)	0
Social Supports	2 (14, 19)	0
Number of Abnormal GA Domains	1 (14)	0
VES-13 Score	1 (16)	0

Note: (a) The definition of treatment plan modification and the way each predictor variable is assessed/defined in these studies is not necessarily the same as in the present study. (b) For all studies, the p-value threshold for significance was <0.05, except Chaïbi et al. (2011), which used a p-value threshold for significance of <0.01. (c) The numbers in parentheses under the univariable and multivariable columns are the reference numbers from the main text for the papers in which the indicated result was found.

Supplementary Table S2. Descriptions of the evaluation methods for each GA domain and what constitutes an abnormal designation in our study.

GA Domain	Evaluation Method(s)	Abnormal Designation
**Cognition**	• History<br>• Mini-Cog Test	• Abnormal features on history (based on clinical judgement) and/or a positive screen for dementia on the Mini-Cog Test (a score of 0-2)
**Comorbidities**	• Charlson Comorbidity Index (CCI) plus clinician judgement	• A moderate (2-3) or high (≥4) score
**Falls Risk**	• History	• ≥1 falls in the last 6 months
**Functional Status**	• Instrumental Activities of Daily Living (IADLs) scale<br>• Physical performance measures (grip strength and the Short Physical Performance Battery)	• Dependence in ≥1 IADL and/or an abnormal performance in either physical performance measure (using sex-specific normative values for grip strength)
**Medication Optimization**	• History	• The identification of one or more medications that require(s) addition, cessation, or dose modification
**Mood**	• Patient Health Questionnaire-2 (PHQ-2) and PHQ-9 (the PHQ-2 is used first as a screen, then those who screen positive receive the PHQ-9)[a]	• A PHQ-9 score suggestive of at least mild depression (≥5)
**Nutrition**	• History (including amount of weight loss)<br>• Body mass index (BMI)<br>• Albumin levels	• At-risk for malnourishment (in general, mild weight loss, normal albumin, and/or decreased appetite) or malnourished (in general, clinically significant weight loss [loss of 4-5% or more within one year or 10% or more within any period] or newly low BMI [<20], low albumin [<35-36 g/L], and/or history of decreased appetite)
**Social Supports**	• History	• History indicating vulnerability or no social supports (based on clinical judgement)

Note: (a) The PHQ-2 was introduced during the study period (prior to that point, every patient received the PHQ-9).

Supplementary Table S3. Descriptions of the types of treatment plan impact.

Treatment Plan Impact	Description
**Increased in intensity**	Final (post-geriatric assessment [GA]) treatment more intensive than the initial treatment either in terms of modality (e.g., radiation to surgery, single mode to multimodal), number of drugs (single agent to multi-agent chemotherapy), or dose (from dose-reduced to full-dose).
**Unchanged**	Initial and final (post-GA) treatment plans were unchanged.
**Decreased in intensity**	Final (post-GA) treatment less intensive than the initial treatment, either in terms of modality (e.g., surgery to radiation, multimodal to single mode), number of drugs (multi-agent to single agent chemotherapy), or dose (from full dose to dose-reduced).
**Changed to supportive care only**	Initial treatment included either curative or palliative intent surgery, radiation, or systemic therapy, whereas final (post-GA) plan was for supportive care alone.
**Deferred**	Treatment was deferred by at least 7 days based on either clinical grounds or patient preference.
**Other**	Various reasons, including change in disease stage, patient death before treatment initiation, pathology not showing cancer, etc.

Supplementary Table S4. Complete results of univariable analysis examining the relationship between various predictor variables and treatment plan modification.

Predictor Variable		OR (95% CI)	p-value	AUC
**Age (Categorical)**	**Overall**	N/A	0.064	0.567
	**61-70 (reference)**	N/A	N/A	
	**71-75**	1.00 (0.51-1.93)	0.991	
	**76-80**	1.53 (0.81-2.89)	0.193	
	**81-85**	1.30 (0.70-2.41)	0.411	
	**86-90**	1.84 (0.96-3.51)	0.66	
	**91-100**	2.35 (0.98-5.64)	0.56	
**Age (Continuous)**		1.03 (1.01-1.06)	0.003*	0.563
**Sex (Male)**		0.90 (0.68-1.21)	0.489	0.513
**VES-13 Score (Abnormal, ≥3)**		3.35 (2.29-4.91)	<0.001*	0.597
**CARG Toxicity Risk**	**Overall**	N/A	<0.001*	0.649
	**Low (reference)**	N/A	N/A	
	**Moderate**	2.10 (1.09-4.05)	0.027*	
	**High**	5.86 (2.97-11.56)	<0.001*	
	**N/A[(b)]**	1.35 (0.72-2.52)	0.356	
**Disease Site**	**Overall**	N/A	0.799	0.530
	**Gastrointestinal (reference)**	N/A	N/A	
	**Head & Neck**	0.83 (0.54-1.27)	0.385	
	**Genitourinary**	1.08 (0.69-1.68)	0.740	
	**Gynecological**	1.12 (0.65-1.92)	0.680	
	**Lymphoma**	1.18 (0.69-2.00)	0.552	
	**Other**	1.09 (0.68-1.74)	0.717	
**Disease Stage**	**Overall**	N/A	<0.001*	0.588
	**Localized (reference)**	N/A	N/A	
	**Biochemical Relapse or Locally Advanced**	1.77 (1.23-2.55)	0.002*	
	**Metastatic**	2.22 (1.48-3.32)	<0.001*	
	**Unknown**	11.53 (1.42-93.72)	0.022*	
	**N/A[(c)]**	1.70 (1.03-2.81)	0.038*	
**Treatment Stage**	**Overall**	N/A	0.009*	0.548
	**Active Surveillance (reference)**	N/A	N/A	
	**Pre-Treatment**	Not specified	1.00	
	**Pre-Treatment, New Modality**	Not specified	1.00	
**Treatment Intent**	**Overall**	N/A	<0.001*	0.590
	**Palliative (reference)**	N/A	N/A	
	**Curative or Neo/Adjuvant**	0.48 (0.35-0.66)	<0.001*	
	**Unknown**	1.46 (0.55-3.89)	0.448	
**Cognition (Abnormal)**		2.24 (1.57-3.19)	<0.001*	0.572
**Comorbidities (Moderate/High)**		2.16 (1.61-2.91)	<0.001*	0.594

**Falls Risk (Increased)**		2.49 (1.83-3.37)	<0.001*	0.606
**Functional Status (Impaired Physical Performance and/or IADLs)**		2.69 (1.89-3.83)	<0.001*	0.588
**Medication Optimization (Potential for Optimization)**		1.10 (0.81-1.51)	0.537	0.511
**Mood (Depressed)**		1.62 (1.05-2.50)	0.028*	0.529
**Nutrition (At-Risk/Malnourished)**		1.39 (1.04-1.85)	0.028*	0.541
**Social Supports (Vulnerable/None)**		1.10 (0.79-1.54)	0.570	0.509
**Number of Abnormal Domains**	**Overall**	N/A	<0.001*	0.660
	**0 (reference)**	N/A	N/A	
	**1**	0.732 (0.23-2.30)	0.593	
	**2**	1.11 (0.38-3.24)	0.853	
	**3**	1.45 (0.51-4.07)	0.485	
	**4**	2.66 (0.96-7.39)	0.061	
	**5**	3.92 (1.39-11.08)	0.010*	
	**6**	4.95 (1.64-14.93)	0.004*	
	**7**	4.44 (1.27-15.62)	0.020*	
	**8**	2.00 (0.22-17.89)	0.535	
**Threshold of 1 (≥1 Abnormal Domains)**		2.24 (0.83-6.02)	0.111	0.509
**Threshold of 2 (≥2 Abnormal Domains)**		3.31 (1.82-5.23)	<0.001*	0.548
**Threshold of 3 (≥3 Abnormal Domains)**		2.88 (1.99-4.17)	<0.001*	0.588
**Threshold of 4 (≥4 Abnormal Domains)**		2.94 (2.17-3.99)	<0.001*	0.628
**Threshold of 5 (≥5 Abnormal Domains)**		2.62 (1.90-3.61)	<0.001*	0.604
**Threshold of 6 (≥6 Abnormal Domains)**		2.37 (1.52-3.69)	<0.001*	0.551
**Threshold of 7 (≥7 Abnormal Domains)**		1.87 (0.90-3.92)	0.096	0.513
**Threshold of 8 (8 Abnormal Domains)**		0.91 (0.13-6.50)	0.926	0.500

Note: (a) "VES-13" = Vulnerable Elders Survey-13, "CARG" = Cancer and Aging Research Group, * = statistically significant p-value (<0.050), OR = odds ratio, CI = confidence interval, and AUC = area under the curve. (b) The "N/A" category under CARG toxicity risk is for patients who were not assigned a CARG risk score as chemotherapy was not planned. (c) The "N/A" category under disease stage is for patients who have a hematologic malignancy and, therefore, the conventional staging system used for solid tumours does not apply.

Supplementary Table S5. Complete results of multivariable analysis examining the relationship between various predictor variables and treatment plan modification.

No.	Predictor Variable		OR (95% CI)	OR p-value	AUC	HL
1	Age (Continuous)		1.04 (1.02-1.07)	0.001*	0.704	0.409
	Sex (Male)		0.84 (0.60-1.16)	0.280		
	VES-13 Score (Abnormal, ≥3)		-	-		
	Treatment Intent	Palliative (reference)	N/A	N/A		
		Curative or Neo/Adjuvant	0.58 (0.41-0.82)	0.002*		
		Unknown	1.32 (0.43-4.08)	0.626		
	Cognition (Abnormal)		1.67 (1.13-2.47)	0.010*		
	Comorbidities (Moderate/High)		1.90 (1.36-2.65)	<0.001*		
	Falls Risk (Increased)		1.96 (1.40-2.76)	<0.001*		
	Mood (Depressed)		1.36 (0.84-2.19)	0.209		
	Nutrition (At-Risk/Malnourished)		1.31 (0.94-1.82)	0.115		
2	Age (Continuous)		1.03 (1.00-1.05)	0.038*	0.689	0.348
	Sex (Male)		0.98 (0.72-1.35)	0.920		
	VES-13 Score (Abnormal, ≥3)		2.01 (1.31-3.09)	0.001*		
	Treatment Intent	Palliative (reference)	N/A	N/A		
		Curative or Neo/Adjuvant	0.56 (0.40-0.77)	<0.001*		
		Unknown	1.36 (0.50-3.68)	0.549		
	Threshold of 4 (≥4 Abnormal Domains)		2.29 (1.64-3.20)	<0.001*		
3	Age (Continuous)		1.02 (0.99-1.05)	0.097	0.679	0.723
	Sex (Male)		0.89 (0.65-1.22)	0.483		
	VES-13 Score (Abnormal, ≥3)		2.57 (1.71-3.85)	<0.001*		
	Treatment Intent	Palliative (reference)	N/A	N/A		
		Curative or Neo/Adjuvant	0.53 (0.38-0.74)	<0.001*		
		Unknown	1.37 (0.46-4.06)	0.567		
	Cognition (Abnormal)		1.96 (1.35-2.83)	<0.001*		
4	Age (Continuous)		1.02 (0.99-1.05)	0.086	0.679	0.522

	**Sex (Male)**		0.90 (0.66-1.23)	0.520		
	**VES-13 Score (Abnormal, ≥3)**		2.58 (1.72-3.88)	<0.001*		
	**Treatment Intent**	**Palliative (reference)**	N/A	N/A		
		**Curative or Neo/Adjuvant**	0.54 (0.39-0.75)	<0.001*		
		**Unknown**	1.18 (0.43-3.22)	0.746		
	**Comorbidities (Moderate/High)**		1.88 (1.38-2.58)	<0.001*		
**5**	**Age (Continuous)**		1.02 (0.99-1.05)	0.060	0.678	0.507
	**Sex (Male)**		0.99 (0.73-1.35)	0.956		
	**VES-13 Score (Abnormal, ≥3)**		2.23 (1.46-3.39)	<0.001*		
	**Treatment Intent**	**Palliative (reference)**	N/A	N/A		
		**Curative or Neo/Adjuvant**	0.53 (0.39-0.74)	<0.001*		
		**Unknown**	1.40 (0.52-3.79)	0.511		
	**Falls Risk (Increased)**		1.97 (1.42-2.74)	<0.001*		
**6**	**Age (Continuous)**		1.03 (1.01-1.06)	0.006*	0.662	0.302
	**Sex (Male)**		0.92 (0.68-1.25)	0.606		
	**VES-13 Score (Abnormal, ≥3)**		-	-		
	**Treatment Intent**	**Palliative (reference)**	N/A	N/A		
		**Curative or Neo/Adjuvant**	0.50 (0.36-0.69)	<0.001*		
		**Unknown**	1.85 (0.64-5.35)	0.257		
	**Functional Status (Impaired Physical Performance and/or IADLs)**		2.57 (1.79-3.70)	<0.001*		
**7**	**Age (Continuous)**		1.02 (0.99-1.05)	0.082	0.650	0.834
	**Sex (Male)**		1.01 (0.74-1.39)	0.933		
	**VES-13 Score (Abnormal, ≥3)**		2.73 (1.82-4.12)	<0.001*		
	**Treatment Intent**	**Palliative (reference)**	N/A	N/A		
		**Curative or Neo/Adjuvant**	0.56 (0.40-0.78)	<0.001*		
		**Unknown**	1.13 (0.40-3.16)	0.821		
	**Mood (Depressed)**		1.45 (0.91-2.23)	0.116		
**8**	**Age (Continuous)**		1.02 (0.99-1.04)	0.113	0.660	0.842

	**Sex (Male)**		0.96 (0.71-1.31)	0.808		
	**VES-13 Score (Abnormal, ≥3)**		2.77 (1.85-4.15)	<0.001*		
	**Treatment Intent**	**Palliative (reference)**	N/A	N/A		
		**Curative or Neo/Adjuvant**	0.53 (0.38-0.73)	<0.001*		
		**Unknown**	1.34 (0.50-3.61)	0.566		
	**Nutrition (At-Risk/Malnourished)**		1.24 (0.91-1.68)	0.177		
**9**	**Age (Continuous)**		1.03 (1.00-1.05)	0.027*	0.700	0.771
	**Sex (Male)**		0.92 (0.67-1.26)	0.596		
	**VES-13 Score (Abnormal, ≥3)**		1.90 (1.23-2.93)	0.004*		
	**Treatment Intent**	**Palliative (reference)**	N/A	N/A		
		**Curative or Neo/Adjuvant**	0.56 (0.40-0.78)	<0.001*		
		**Unknown**	1.44 (0.48-4.26)	0.513		
	**Threshold of 4 (≥4 Abnormal Domains)**		2.10 (1.47-3.00)	<0.001*		
	**Cognition (Abnormal)**		1.51 (1.02-2.23)	0.040*		
**10**	**Age (Continuous)**		1.03 (1.00-1.05)	0.033*	0.699	0.927
	**Sex (Male)**		0.93 (0.68-1.28)	0.671		
	**VES-13 Score (Abnormal, ≥3)**		1.98 (1.28-3.04)	0.002*		
	**Treatment Intent**	**Palliative (reference)**	N/A	N/A		
		**Curative or Neo/Adjuvant**	0.56 (0.41-0.78)	<0.001*		
		**Unknown**	1.27 (0.46-3.46)	0.646		
	**Threshold of 4 (≥4 Abnormal Domains)**		1.99 (1.40-2.83)	<0.001*		
	**Comorbidities (Moderate/High)**		1.54 (1.11-2.15)	0.010*		
**11**	**Age (Continuous)**		1.03 (1.00-1.05)	0.032*	0.691	0.111
	**Sex (Male)**		0.99 (0.72-1.35)	0.945		
	**VES-13 Score (Abnormal, ≥3)**		1.92 (1.24-2.95)	0.003*		
	**Treatment Intent**	**Palliative (reference)**	N/A	N/A		
		**Curative or Neo/Adjuvant**	0.55 (0.40-0.77)	<0.001*		
		**Unknown**	1.38 (0.51-3.76)	0.523		
	**Threshold of 4 (≥4 Abnormal Domains)**		1.91 (1.28-2.84)	0.001*		

	**Falls Risk (Increased)**		1.40 (0.94-2.06)	0.104		
**12**	**Age (Continuous)**		1.04 (1.01-1.06)	0.003*	0.689	0.295
	**Sex (Male)**		0.94 (0.69-1.28)	0.695		
	**VES-13 Score (Abnormal, ≥3)**		-	-		
	**Treatment Intent**	**Palliative (reference)**	N/A	N/A		
		**Curative or Neo/Adjuvant**	0.53 (0.39-0.74)	<0.001*		
		**Unknown**	1.79 (0.61-5.24)	0.289		
	**Threshold of 4 (≥4 Abnormal Domains)**		2.35 (1.62-3.40)	<0.001*		
	**Functional Status (Impaired Physical Performance and/or IADLs)**		1.51 (0.98-2.33)	0.06		
**13**	**Age (Continuous)**		1.03 (1.00-1.05)	0.028*	0.685	0.525
	**Sex (Male)**		1.02 (0.74-1.40)	0.911		
	**VES-13 Score (Abnormal, ≥3)**		1.97 (1.27-3.05)	0.002*		
	**Treatment Intent**	**Palliative (reference)**	N/A	N/A		
		**Curative or Neo/Adjuvant**	0.59 (0.42-0.82)	0.002*		
		**Unknown**	1.22 (0.43-3.43)	0.707		
	**Threshold of 4 (≥4 Abnormal Domains)**		2.20 (1.56-3.11)	<0.001*		
	**Mood (Depressed)**		1.16 (0.72-1.86)	0.540		
**14**	**Age (Continuous)**		1.03 (1.00-1.05)	0.037*	0.689	0.858
	**Sex (Male)**		0.98 (0.72-1.34)	0.887		
	**VES-13 Score (Abnormal, ≥3)**		1.98 (1.29-3.04)	0.002*		
	**Treatment Intent**	**Palliative (reference)**	N/A	N/A		
		**Curative or Neo/Adjuvant**	0.55 (0.40-0.76)	<0.001*		
		**Unknown**	1.34 (0.49-3.64)	0.567		
	**Threshold of 4 (≥4 Abnormal Domains)**		2.31 (1.63-3.29)	<0.001*		
	**Nutrition (At-Risk/Malnourished)**		0.97 (0.69-1.34)	0.834		

Note: (a) "*" = statistically significant p-value (<0.050), "OR" = odds ratio, "CI" = confidence interval, "AUC" = area under the curve, and "HL" = Hosmer-Lemeshow statistic p-value. (b) OR values were rounded up to report to 2 decimal places (unless value would round to 1.00, in which case the OR was kept at 0.99 to show that the 95% CI crosses 1.00). (c) Reference categories for each variable are the same as noted in Table 2.

Supplementary Table S6. Complete results of multivariable analysis examining the relationship between various predictor variables and treatment plan modification (sensitivity analysis, excluding VES-13 score).

No.	Predictor Variable		OR (95% CI)	OR p-value	AUC	HL
2	Age (Continuous)		1.04 (1.01-1.06)	0.002*	0.682	0.676
	Sex (Male)		0.94 (0.69-1.28)	0.708		
	Treatment Intent	Palliative (reference)	N/A	N/A		
		Curative or Neo/Adjuvant	0.536 (0.39-0.74)	<0.001*		
		Unknown	1.44 (0.53-3.94)	0.474		
	Threshold of 4 (≥4 Abnormal Domains)		2.81 (2.06-3.84)	<0.001*		
3	Age (Continuous)		1.03 (1.01-1.06)	0.005*	0.657	0.545
	Sex (Male)		0.82 (0.61-1.12)	0.210		
	Treatment Intent	Palliative (reference)	N/A	N/A		
		Curative or Neo/Adjuvant	0.49 (0.36-0.68)	<0.001*		
		Unknown	1.46 (0.50-4.27)	0.493		
	Cognition (Abnormal)		2.16 (1.50-3.11)	<0.001*		
4	Age (Continuous)		1.04 (1.01-1.06)	0.003*	0.659	0.444
	Sex (Male)		0.83 (0.61-1.12)	0.220		
	Treatment Intent	Palliative (reference)	N/A	N/A		
		Curative or Neo/Adjuvant	0.51 (0.37-0.70)	<0.001*		
		Unknown	1.24 (0.46-3.35)	0.672		
	Comorbidities (Moderate/High)		2.12 (1.56-2.87)	<0.001*		
5	Age (Continuous)		1.04 (1.01-1.06)	0.003*	0.669	0.711
	Sex (Male)		0.94 (0.70-1.28)	0.708		
	Treatment Intent	Palliative (reference)	N/A	N/A		
		Curative or Neo/Adjuvant	0.51 (0.37-0.70)	<0.001*		
		Unknown	1.52 (0.56-4.15)	0.411		
	Falls Risk (Increased)		2.42 (1.77-3.31)	<0.001*		
7	Age (Continuous)		1.04 (1.01-1.06)	0.002*	0.619	0.686

	Sex (Male)		0.93 (0.68-1.27)	0.645		
	Treatment Intent	Palliative (reference)	N/A	N/A		
		Curative or Neo/Adjuvant	0.52 (0.38-0.72)	<0.001*		
		Unknown	1.21 (0.43-3.36)	0.717		
	Mood (Depressed)		1.58 (1.01-2.48)	0.045*		
8	Age (Continuous)		1.04 (1.01-1.06)	0.003*	0.635	0.744
	Sex (Male)		0.89 (0.66-1.20)	0.428		
	Treatment Intent	Palliative (reference)	N/A	N/A		
		Curative or Neo/Adjuvant	0.49 (0.36-0.68)	<0.001*		
		Unknown	1.46 (0.54-3.92)	0.453		
	Nutrition (At-Risk/Malnourished)		1.33 (0.99-1.80)	0.062		
9	Age (Continuous)		1.04 (1.01-1.06)	0.002*	0.693	0.883
	Sex (Male)		0.88 (0.64-1.21)	0.431		
	Treatment Intent	Palliative (reference)	N/A	N/A		
		Curative or Neo/Adjuvant	0.54 (0.39-0.75)	<0.001*		
		Unknown	1.52 (0.51-4.52)	0.456		
	Threshold of 4 (≥4 Abnormal Domains)		2.56 (1.83-3.59)	<0.001*		
	Cognition (Abnormal)		1.50 (1.01-2.20)	0.046*		
10	Age (Continuous)		1.04 (1.01-1.06)	0.002*	0.692	0.593
	Sex (Male)		0.89 (0.65-1.22)	0.475		
	Treatment Intent	Palliative (reference)	N/A	N/A		
		Curative or Neo/Adjuvant	0.55 (0.39-0.76)	<0.001*		
		Unknown	1.33 (0.49-3.65)	0.577		
	Threshold of 4 (≥4 Abnormal Domains)		2.41 (1.73-3.36)	<0.001*		
	Comorbidities (Moderate/High)		1.58 (1.14-2.20)	0.006*		
11	Age (Continuous)		1.04 (1.01-1.06)	0.002*	0.686	0.730
	Sex (Male)		0.95 (0.70-1.30)	0.762		
	Treatment Intent	Palliative (reference)	N/A	N/A		

		**Curative or Neo/Adjuvant**	0.53 (0.39-0.74)	<0.001*		
		**Unknown**	1.47 (0.52-4.02)	0.449		
	**Threshold of 4 (≥4 Abnormal Domains)**		2.20 (1.49-3.23)	<0.001*		
	**Falls Risk (Increased)**		1.51 (1.02-2.24)	0.038*		
**13**	**Age (Continuous)**		1.04 (1.01-1.06)	0.002*	0.677	0.887
	**Sex (Male)**		0.97 (0.71-1.33)	0.846		
	**Treatment Intent**	**Palliative (reference)**	N/A	N/A		
		**Curative or Neo/Adjuvant**	0.56 (0.40-0.78)	<0.001*		
		**Unknown**	1.30 (0.46-3.66)	0.626		
	**Threshold of 4 (≥4 Abnormal Domains)**		2.66 (1.92-3.69)	<0.001*		
	**Mood (Depressed)**		1.17 (0.73-1.87)	0.508		
**14**	**Age (Continuous)**		1.04 (1.01-1.06)	0.002*	0.683	0.943
	**Sex (Male)**		0.94 (0.69-1.27)	0.675		
	**Treatment Intent**	**Palliative (reference)**	N/A	N/A		
		**Curative or Neo/Adjuvant**	0.53 (0.38-0.73)	<0.001*		
		**Unknown**	1.42 (0.52-3.87)	0.498		
	**Threshold of 4 (≥4 Abnormal Domains)**		2.84 (2.04-3.96)	<0.001*		
	**Nutrition (At-Risk/Malnourished)**		0.95 (0.69-1.32)	0.760		

Note: (a) “*” = statistically significant p-value (<0.050), “OR” = odds ratio, “CI” = confidence interval, “AUC” = area under the curve, and “HL” = Hosmer-Lemeshow statistic p-value. (b) OR values were rounded up to report to 2 decimal places (unless value would round to 1.00, in which case the OR was kept at 0.99 to show that the 95% CI crosses 1.00). (c) Reference categories for each variable are the same as noted in Table 2.
